# Meta-analysis of the correlation between dietary copper supply and broiler performance

**DOI:** 10.1371/journal.pone.0232876

**Published:** 2020-05-18

**Authors:** Chao Feng, Bin Xie, Qiqige Wuren, Minghua Gao

**Affiliations:** 1 Department of Life Sciences, Hulunbuir University, Hulunbuir, Inner Mongolia Autonomous Region, China; 2 Department of Agriculture and Forestry, Hulunbuir University, Hulunbuir, Inner Mongolia Autonomous Region, China; Tokat Gaziosmanpasa University, TURKEY

## Abstract

**Objective:**

To conduct a meta-analysis assessing the correlation between dietary copper supply and broiler performance

**Methods:**

Studies that were published prior to January 2019 and reported the dietary copper supply and broiler growth performance were identified using search functions in the Web of Science, Springer, Elsevier, Science Direct, and Taylor & Francis Online databases; the Journal of Dairy Research; and China National Knowledge Infrastructure (CNKI). We performed stratified analyses on the possible sources of bias, including differences in the study locations and years of publication. The publication bias was assessed with Egger’s test method.

**Results:**

A total of 12 randomized controlled trial (RCT) studies were eligible for inclusion. The pooled WMDs of the ADG, ADFI and FCR were -0.166 (95% CI: -1.587 to 1.254), -0.844 (95% CI: -1.536 to -0.152) and -0.029 (95% CI: -0.057 to 0.000), respectively. In the Israeli and Indian studies, the ADG and ADFI data in the experimental group were higher than those in the control group; however, in America, a relatively high FCR value was found in the experimental group compared to that in the control group. The analysis of the study period showed that for the 1980s and 2010s, the ADG and ADFI of the experimental group were lower than those of the control group, while, in the 1990s and 2010s, the FCR of the experimental group were lower than those of the control group. The observed values were adjusted for study effects, and a model was used to obtain the copper supplementation under the optimal production performance. The results showed that the adjusted average daily gain (ADG), average daily feed intake (ADFI), and feed to gain ratio (FCR) presented a quadratic relationship with Cu supplementation (P<0.05). The maximum value of ADG (31.84 g/d) is reached when Cu is added at amount of 158 mg/kg, and the minimum value of FCR (1.53) is reached when Cu is added at amount of 217 mg/kg. No significant publication bias existed in the studies (Egger's test: *P* value were 0.81, 0.71 and 0.14).

**Conclusion:**

From this study, it can be concluded that the traditional copper addition is no longer suitable for modern broiler breeding; the higher copper content may be beneficial for the production performance of broilers.

## 1. Introduction

Copper plays a vital role in the growth of broilers, having significant influences on the maintenance of production performance and regulation of enzymatic activity and on bone growth, glucose metabolism, hemoglobin synthesis, cardiovascular structural integrity, and other physiological functions [1]. According to NRC (1994), the dietary copper requirement of broilers is 8 mg/kg [2], and a higher dose is often given in the production process to obtain better economic benefits yet [3]. It cannot be ignored that excessive copper addition, which occurs because of the cost-effectiveness and easy availability, also causes environmental pollution and waste. Today, the optimum addition of dietary copper is still controversial. Therefore, the level of copper addition urgently needs to be reassessed. Studies have shown that with increasing copper level (100–300 mg/kg), the live performance of broilers is improved [4–6]. Some researchers suggested that feeding 250 ppm of Cu during the starter period had negative consequences on bird performance, and moderate levels of Cu (125 ppm) had beneficial effects on bird performance at market age [7]. Based on studies of the relationship between high levels of dietary copper (100–300 mg/kg) and the performance of broilers, it has been suggested that Cu had an apparent inhibitory effect on the cholesterol content, which can significantly improve the survival rate of broiler offspring [8–9]. In addition, copper oversupply (greater than 300 mg/kg) may inhibit the growth of broilers [10]. Meta-analysis is a kind of statistical method that integrates research results of all types in the same field. The differences between studies are removed by meta-analysis, which can make the corrected data comparable, creating more objective and convincing data [11]. The main goal of this study is to explore the relationship between copper addition and broiler performance reported by different studies through meta-analysis and obtain a reasonable quantitative model to explain the observed value and provide a theoretical reference for the practical process of Cu addition.

## 2. Materials and methods

### 2.1. Dataset

All kinds of randomized controlled trial (RCT) studies published up to January 2019 were retrieved from the Web of Science, Springer, Elsevier, ScienceDirect, and Taylor & Francis Online databases, the Journal of Dairy Research and China National Knowledge Infrastructure (CNKI). The following search terms were used: (broiler or chick*) and (performance or growth) and copper We used broiler, performance, and copper as the keywords to search in the databases listed above, not including papers in conference proceedings and unpublished results. The selected studies met the following criteria: they were published in English or Chinese, used a corn and soybean meal-based diet, had a 21-day experimental period, provided the specific Cu addition values (inorganic), and pertained to non-indigenous breeds. Newborn broilers are divided into groups based on the copper supply they receive. In the control group, the copper supply was zero, and the copper supply in the experimental group ranged from 4 mg/kg to 800 mg/kg. During a 21-day period, the weight changes in the broilers (the initial and final weight are provided in some studies) and food intake were measured daily, and the ADG and ADFI could be calculated from these data. Some studies do not provide the FCR, which can be calculated based on the ADG and ADFI. The data included means, some measure of variance, and at least three independent replicates of each treatment. We extracted the means of the control and experimental treatment (ADG, ADFI and FCR), as well as their standard deviations (SD) and sample sizes (n). When SE was reported, we transformed it to SD by using the formula SD = SE * sqrt (n). If the data were presented graphically, we extracted data points through GetData software (http://www.getdata-graph-digitizer.com/). In total, we collected 12 papers published between 1983 and 2012 (see S1 Table).

### 2.2. Meta-analysis

The PROC MIXED model (SAS Version 9.4, Cary, NC) was used to analyze the relationship between the copper addition level and broiler performance. The model is as follows:

$$Y_{ij}=B_{0}+B_{1}X_{ij}+B_{2}X_{ij}^{2}+S_{i}+b_{1i}X_{ij}+b_{2i}X_{ij}^{2}+e_{ij}$$

where *i* is the number of eligible studies and *j* is the number of observations in each study; *B*_*0*_ is the overall intercept (fixed effects) of all the studies; *B*_*1*_ and *B*_*2*_ are the coefficients of the first and second terms of the polynomial between the different studies (fixed effects), respectively; *X*_*ij*_ is the independent variable of the *j*th observed value in the *i*th study; *S*_*i*_ is the random effect intercept of the *i*th study; *b*_*1i*_ and *b*_*2i*_ are the coefficients of the first and second terms of the polynomial in the *i*th study (random effects), respectively; and *e*_*ij*_ is the residual, which obeys the N (0, σ^2^) distribution (random effects).

The differences between the studies are assumed to be random effects. The intercept and slope of the variables (fixed effects) represent the average intercept and average slope of the ADG, ADFI, and FCR as they vary with dietary copper additions in the mixed effects model. The intercept and slope of the variables (random effects) represent important factors not included in the regression analyses in the different studies [12]. The random effects of the y value are adjusted to remove differences between the studies and then a regression analysis performed to calculate the correlation coefficient (i.e., *r*^*2*^) [13].

The mixed effects model code is shown below.

PROC MIXED data = data;

CLASS Group;

MODEL Y = X X*X/Solution OUTP = Predictionset OUTPM = PredY;

RANDOM intercept X/TYPE = VC SUBJECT = Group SOLUTION;

RUN;

A regression analysis and curve fitting were performed for the relationship between the variables (*y*-axis) and copper addition (*x*-axis). The calculation steps are as follows: (i) calculation of the residual, (ii) calculation of PredY, and (iii) determination of the adjusted *y* value for each independent variable (AdjuestedY = PredY+Residual) to remove the differences between the studies.

We performed meta-analysis of RCTs and reported the pooled weighted mean difference (WMD) with 95% confidence intervals (95% CI) of a total change in ADG, ADFI and FCR. Stratified analysis by geographic areas and published year were also carried out. To assess the effects of each individual study and to verify the stability of the results of the meta-analysis, sensitivity analysis was conducted by removing each study in turn and estimating the overall effect of the remaining studies sequentially. The potential publication bias was evaluated by using Egger's test. All the statistical analyses were performed using STATA (version 11.0; Stata Corp, College Station, TX) and SAS (Version 9.4; SAS Institute, Cary, NC, US) software.

## 3. Results

### 3.1. Database

A total of 12 studies met the above mentioned criteria [14–25]. The studies included in the database are shown in S1 Table. The research is mainly distributed in China (46.2%), the US (30.8%), India (7.7%), Iran (7.7%), and Israel (7.7%).

### 3.2. Results of the meta-analysis

**Fig 1:**
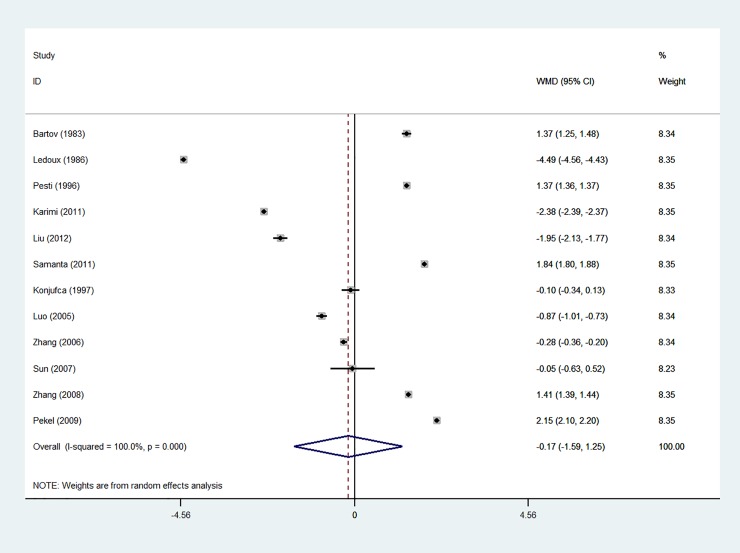
Forest plot of ADG.

**Fig 2:**
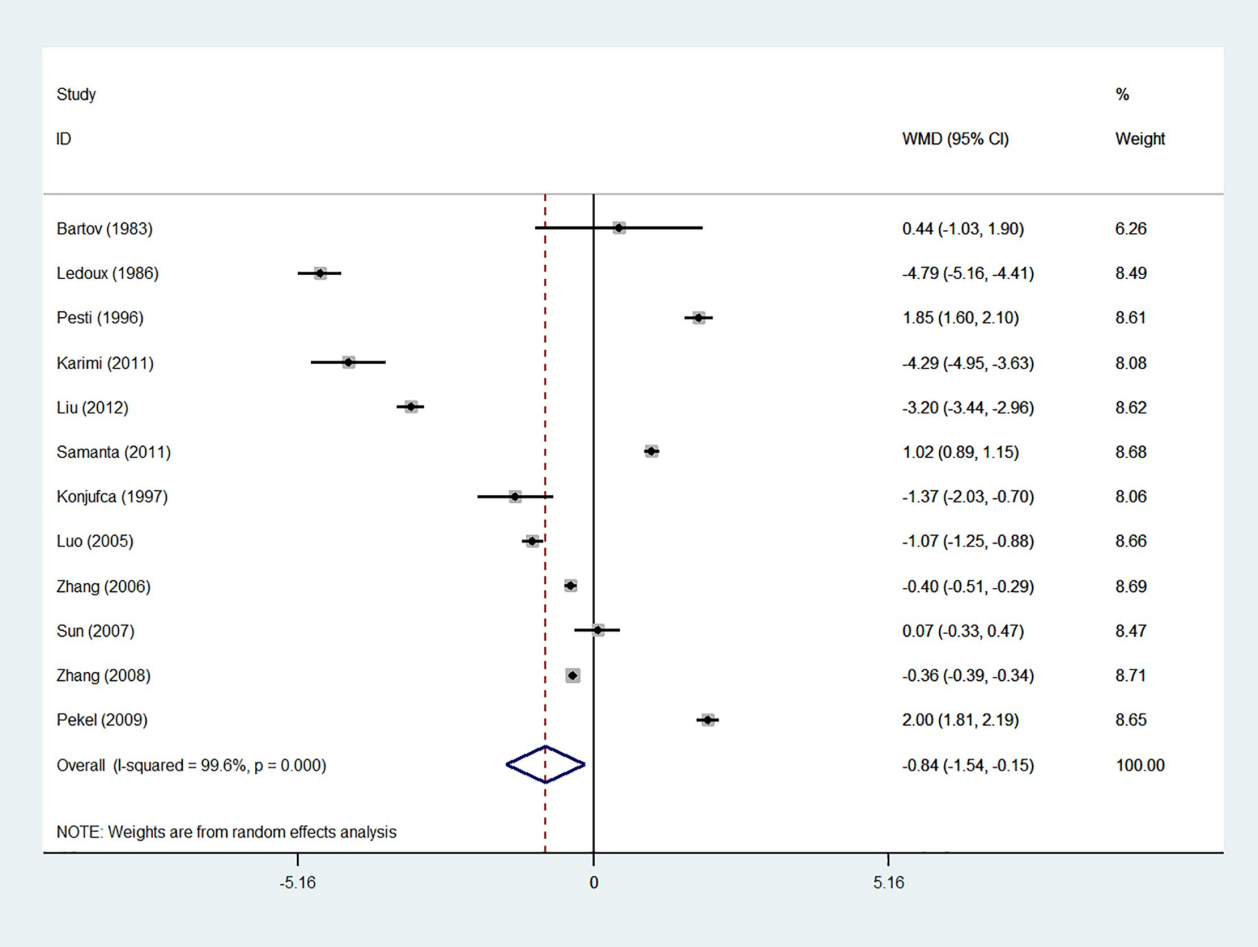
Forest plot of ADFI.

**Fig 3:**
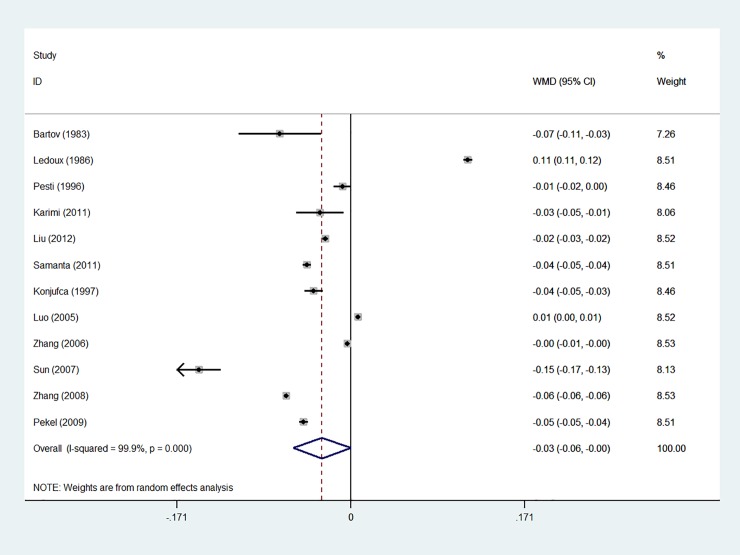
Forest plot of FCR.

Based on the results of the random-effects method, the pooled WMD of the ADG, ADFI and FCR was -0.166 (95% CI: -1.587 to 1.254), -0.844 (95% CI: -1.536 to -0.152) and -0.029 (95% CI: -0.057 to 0.000), respectively. The forest plots are shown in Figs 1–3

### 3.3. Subgroup analyses

The subgroup analysis was stratified by area and showed that the pooled WMD of the ADG was 1.365 (95% CI: 1.246 to 1.484) in Israel, -0.271 (95% CI: -2.605 to 2.063) in the US, -0.381 (95% CI: -2.388 to -2.374) in Iran, -0.348 (95% CI: -1.649 to 0.952) in China, and 1.838 (95% CI: 1.8 to 1.877) in India (Table 1). When stratified by publication years, the pooled WMD of the ADG was -1.565 (95% CI: -7.307 to 4.177) from 1980 to 1990, 0.636 (95% CI: -0.803 to 2.075) from 1990 to 2000, 0.483 (95% CI: -0.348 to 1.315) from 2000 to 2010, and -0.831 (95% CI: -4.134 to 2.473) from 2010 to 2020 (Table 1). The results of subgroup analysis for the ADFI and FCR are detailed in Table 1.

**Table 1:** Stratified analyses by different factors.

	Stratified factors	No. of studies	WMD	95% CI (lower limit)	95% CI (upper limit)	Heterogeneity (I^2^)	Heterogeneity test *P* value	Model
**ADG**								
**Country**								
	Israel	1	1.365	1.246	1.484	-	-	Random
	US	4	-0.271	-2.605	2.063	100%	0	Random
	Iran	1	-0.381	-2.388	-2.374	-	-	Random
	China	5	-0.348	-1.649	0.952	99.9%	0	Random
	India	1	1.838	1.800	1.877	-	-	Random
**Years**								
	1980–1990	2	-1.565	-7.307	4.177	100%	0	Random
	1990–2000	2	0.636	-0.803	2.075	99.3%	0	Random
	2000–2010	5	0.483	-0.348	1.315	99.9%	0	Random
	2010–2020	3	-0.831	-4.134	2.473	100%	0	Random
**ADFI**								
**Country**								
	Israel	1	0.437	-1.03	1.905	-	-	Random
	US	4	-0.572	-3.485	2.341	99.7%	0	Random
	Iran	1	-4.29	-4.946	-3.634	-	-	Random
	China	5	-0.995	-1.67	-0.320	99.9%	0	Random
	India	1	1.017	0.888	1.146	-	-	Random
**Years**								
	1980–1990	2	-2.225	-7.345	2.894	97.8%	0	Random
	1990–2000	2	0.255	-2.894	3.404	98.7%	0	Random
	2000–2010	5	0.045	-0.633	0.724	99.4%	0	Random
	2010–2020	3	-2.149	-5.588	1.290	99.8%	0	Random
**FCR**								
**Country**								
	Israel	1	-0.070	-0.111	-0.029	-	-	Random
	US	4	0.006	-0.086	0.097	99.9%	0	Random
	Iran	1	-0.030	-0.054	-0.007	-	-	Random
	China	5	-0.046	-0.083	-0.008	99.9%	0	Random
	India	1	-0.043	-0.047	-0.039	-	-	Random
**Years**								
	1980–1990	2	0.024	-0.158	0.205	98.7%	0	Random
	1990–2000	2	-0.022	-0.050	0.005	95.1%	0	Random
	2000–2010	5	-0.050	-0.088	-0.012	96.3%	0	Random
	2010–2020	3	-0.033	-0.049	-0.018	99.9%	0	Random

### 3.4. Relationship between performance and Cu addition

**Fig 4:**
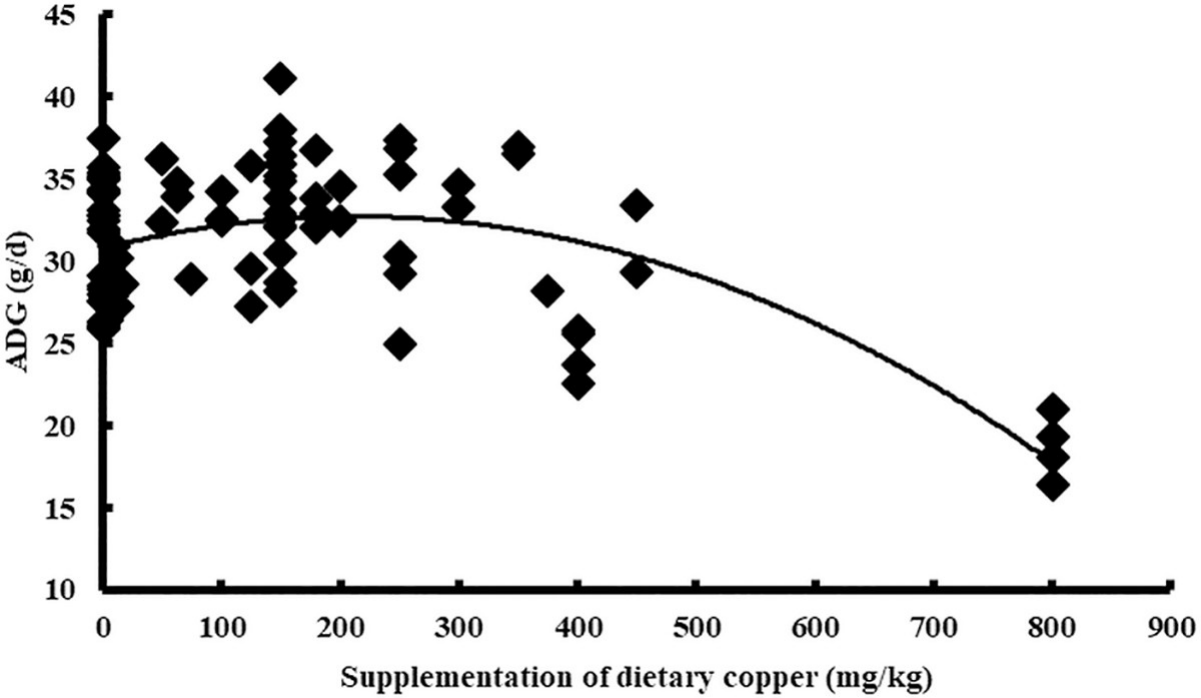
Relationship between copper supplementation and adjusted ADG.

**Fig 5:**
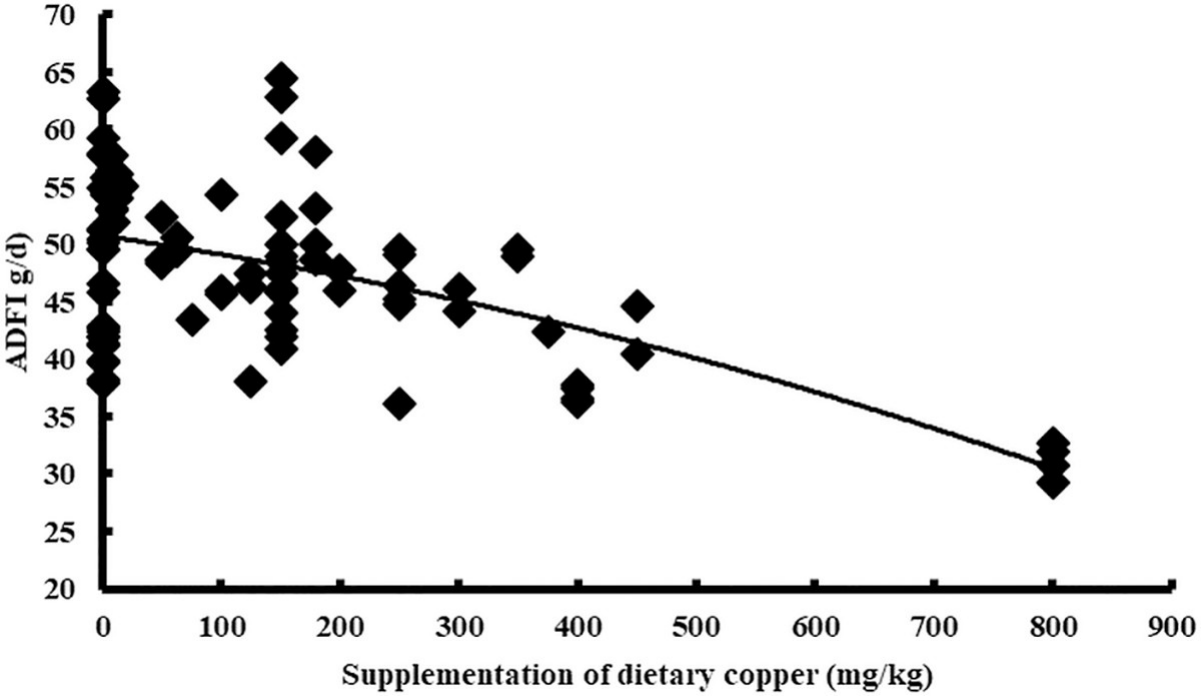
Relationship between copper supplementation and adjusted ADFI.

**Fig 6:**
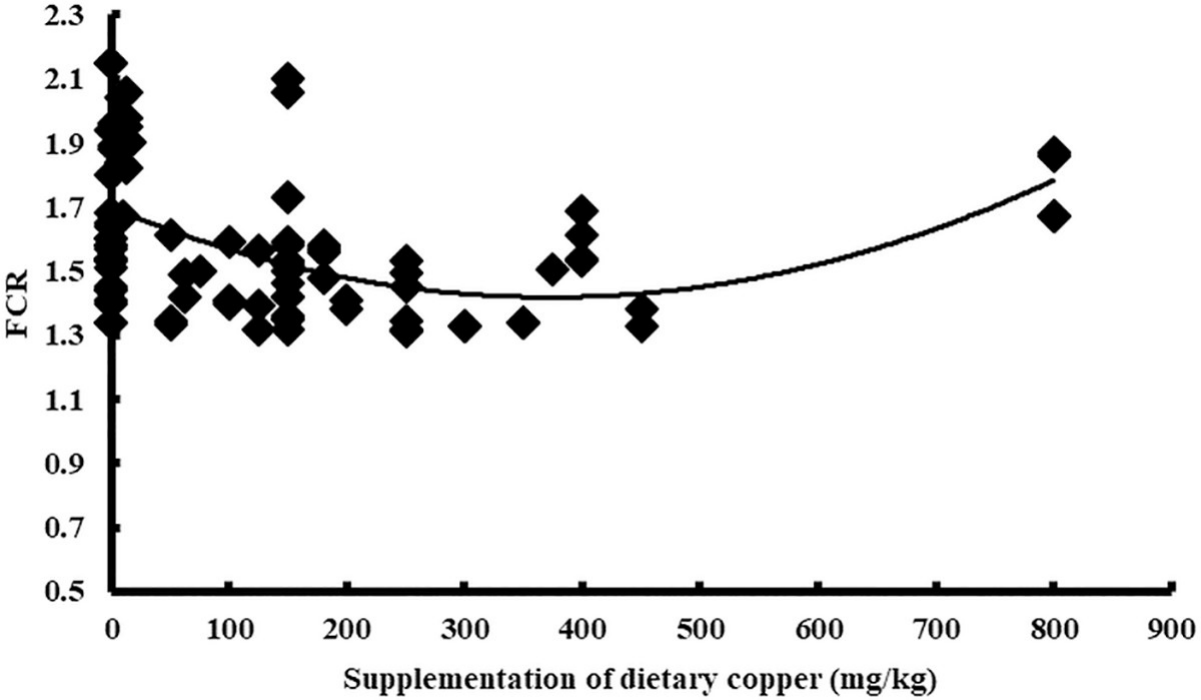
Relationship between copper supplementation and adjusted FCR.

Cu addition was used as the independent variable, and the performance indices (ADG, ADFI, and FCR) of the broiler were used as the dependent variables. The results indicated a quadratic relationship between the adjusted performance index (ADG, ADFI, and FCR) and Cu addition. The maximum values of the ADG (31.84 g/d) and ADFI (47.69 g/d) were reached when Cu addition was 158 mg/kg (Y_ADG_ = 31.49+0.005X-0.00002X^2^, n = 86, P<0.05) and 0 mg/kg (Y_ADFI_ = 47.27–0.00001X^2^, n = 86, P<0.05), respectively. However, the minimum value of the FCR (1.53) was reached when Cu addition was 217 mg/kg (Y_FCR_ = 1.55–0.0003X+0.0000006X^2^, n = 86, *P*<0.05) (Figs 4–6). The statistical results are shown in Table 2.

**Table 2:** Regression relationships inferred using mixed effects models.

Dependent variable	Independent variable	Intercept	SE	*P* value	Quadratic	SE	*P* value	R^2^
ADG	Cu addition	31.49	1.02	<0.05	-2.00E-05	4.18E-06	<0.05	0.54
ADFI		47.27	1.97	<0.05	-1.00E-05	4.68E-06	<0.05	0.55
FCR		1.55	0.06	<0.05	6.12E-07	0	<0.05	0.51

### 3.5. Sensitivity analysis and publication bias

**Fig 7:**
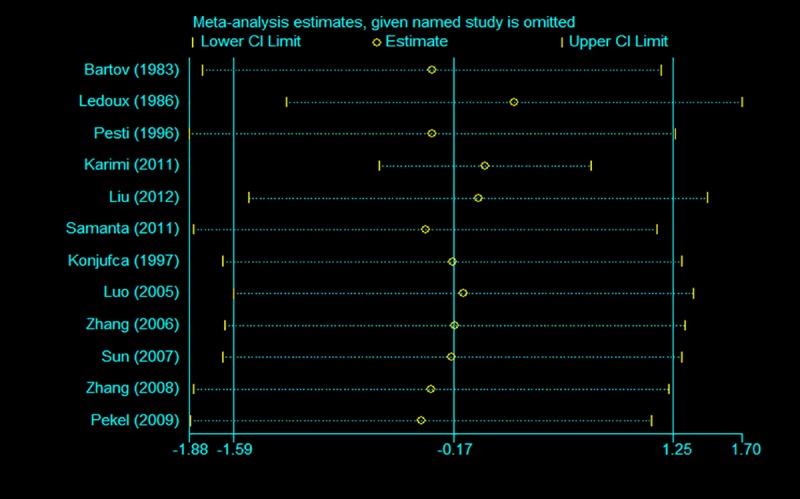
Sensitivity analysis of ADG.

**Fig 8:**
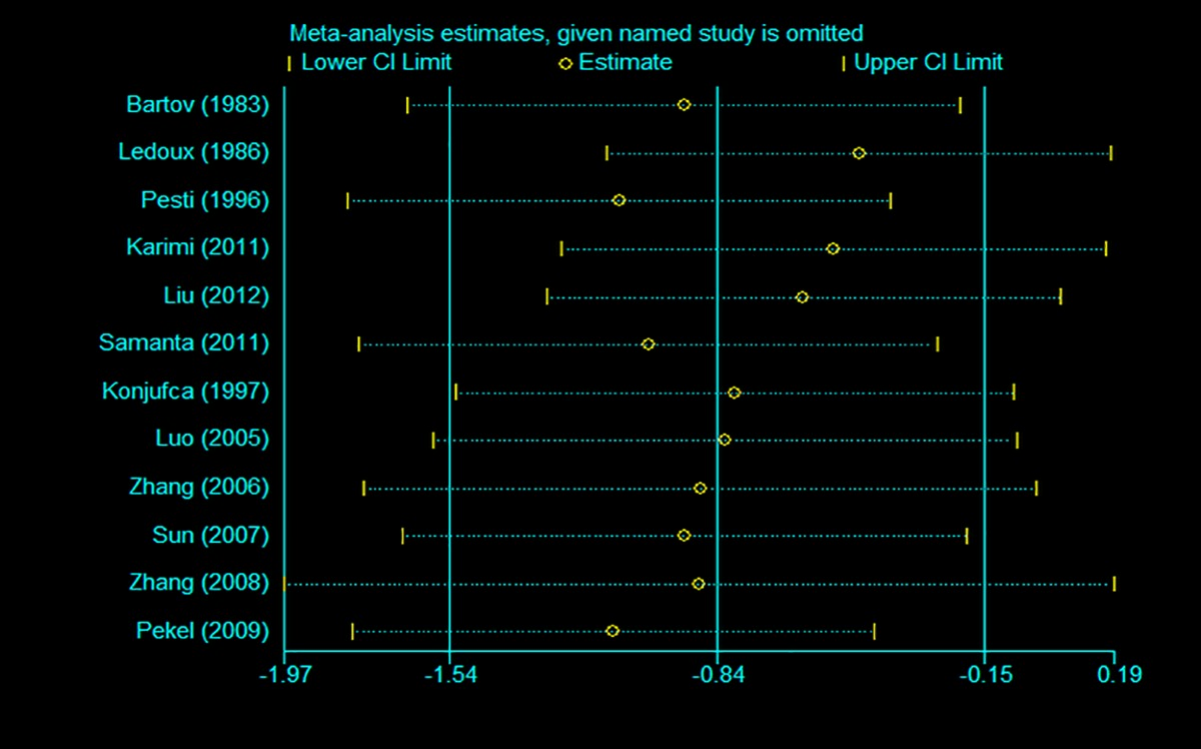
Sensitivity analysis of ADFI.

**Fig 9:**
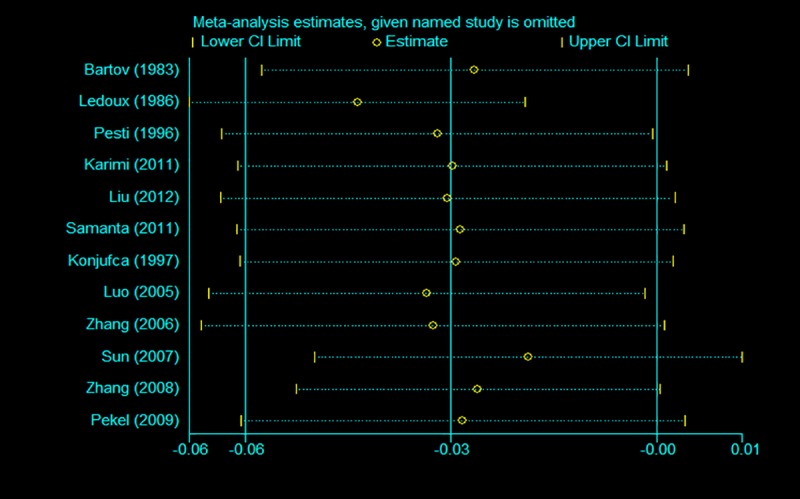
Sensitivity analysis of FCR.

To assess the effects of each individual study and to verify the stability of the results of the meta-analysis, sensitivity analysis was conducted by removing each study in turn and estimating the overall effect of the remaining studies sequentially. After removing each individual study, the pooled estimates of the remaining studies were all located in the range of the overall effect, indicating that the results of the meta-analysis showed low sensitivity and high stability (Figs 7–9). There was little indication of publication bias for studies on the ADG, ADFI or FCR (*P* values for Egger's test were 0.81, 0.71 and 0.14, respectively).

## 4. Discussion

The NRC (1994) recommended 8 mg/kg Cu for broiler diets. However, in practice, an excessive amount of copper was added for a long time. This is because studies have found that increasing the supply of copper in poultry production can effectively improve the live performance of the birds, mainly because of the antibacterial or bacteriostatic properties of copper [17]. In addition, a study on laying hens also showed that an increase in copper addition was effective in changing the lipid composition of eggs and reducing the cholesterol content in eggs [26]. Many studies have also indicated that supplementation in excess of the current recommendations (8 mg/kg) caused growth-promoting effects in broilers, and the copper supplementation at 150 mg/kg was found to have a positive effect on live weight gain in broilers, which might be a consequence of the significant reduction in the total number of pathogenic organisms in the gut [1, 9, 21, 27, 28, 29, 30].

In this study, with the increase in Cu addition, the average daily gain (ADG) of broilers showed a significant negative quadratic relationship, reaching an extreme value when the Cu addition amount was 158 mg/kg (Fig 4). The FCR of the broilers also showed a significant positive quadratic relationship and the lowest FCR value was found when 217 mg/kg of Cu was added (Fig 6). Although there is a quadratic relationship between the ADFI and copper addition (Fig 5), the extreme value of the ADFI is not reached in the interval in which the copper addition is greater than zero, which may be related to a difference in the data. In the modern poultry industry, the amount of copper added has been much higher than 8 mg/kg as the minimum requirement for broiler recommended by NRC. We have also reached a similar conclusion through meta-analysis. To achieve the maximum ADG and the minimum FCR, we believe that the amount of copper added should be 158mg/kg and 217mg/kg, respectively. According to Samanta *et al*., the result was found to be commercially beneficial for the chickens receiving 150mg/kg of diet. There are also studies showed that the addition of 200 or 250mg/kg copper could promote the growth of broilers [19, 28]. These experimental results were considered to have some support for our study. The amount of copper added was often not accurate in the previous studies, most of which only had two or three treatment levels and often showed a multiple relationship between the treatment levels. While meta-analysis integrated the results of various studies, and the final result was more statistically significant. In addition, the copper addition levels obtained in this study are not consistent when the ADG or FCR reach an extremum (maximum or minimum). The addition of copper at 150mg/kg is widely accepted in commercial poultry nutrition, however, we believed that the copper addition might be more appropriate when FCR reached the extreme value. FCR is an important determinant of profitability for broiler producers. Because the feed constitutes 70–80% of the cost of raising broiler chickens, changes in the feed-conversion ratio can have a major impact on the profitability of an operation [31]. Therefore, the recommended copper addition amount is 217mg/kg in this study, but not all studies support this result. According to the positive quadratic relationship between FCR and copper addition, the FCR gradually increased after the copper addition was higher than 217mg/kg. The results of one study suggest that no effect on weight gain and feed conversion even with 500 mg/kg level of copper sulfate in broiler [32], which may indicate that there is not a simple quadratic relationship between copper addition and broiler performance, additionally, more experimental data are needed to support the meta-analysis. In the analysis of different countries, some of the data included only one study; therefore, the result may not be accurate. There was no significant difference between the experimental and control data in the US. For China, while there was no significant change in the ADG data, both the ADFI and FCR declined, suggesting that the copper additive had a positive effect on performance. In the analysis of different years, none of the data showed significant differences, except for the FCR data. From 2000 to now, there was a significant reduction in the FCR, indicating that the use of copper additives has been more accurate in the 21^st^ century, making it possible to avoid the production performance degradation caused by copper contents that are either too high or too low. On the other hand, the rising price of copper has also promoted the broiler industry to seek more reasonable copper additives in the 21^st^ century.

There are several reasons why the addition of Cu in feed can promote the performance of broiler chickens. First, the inhibitory effect of copper on the reproduction of harmful bacteria in the intestines of broilers indirectly promotes the growth process of broilers. Furthermore, copper also controls the microbes in the feed, reducing the microbial consumption of the feed [33]. Second, copper can increase the activities of certain related enzymes, such as GSH-Pox, CuZn-SOD, and intestinal lipase [25]. Third, copper stimulates growth hormone secretion. Yang *et al*. reported that copper reduced hypothalamic somatostatin secretion by regulating catecholamine metabolism [34]. Therefore, the addition of 8mg/kg copper addition is no longer suitable for the broiler breeding industry, and there is currently an urgent need to redefine the amount of Cu needed.

However, our results showed that there was a quadratic relationship between the copper supply and growth performance, which implies that excessive copper addition has certain disadvantages. The remarkable effects of high level of copper sulfate in diets for poultry is the gizzard erosion which means ulceration of the lining of gizzard. It is most likely that the acidic nature of Cu or sulfate dissociated from copper sulfate or even both may be responsible [28]. Growth suppressive effects, which are mainly due to the biological toxicity of excess copper, have been reported in some studies using extremely high amounts of copper additives (>300 ppm) [22,35]. Copper, as a trace element, has a low content in tissues but a high content in the liver, so copper poisoning mainly affects the liver. Typical copper poisoning results in liver cirrhosis, accompanied by hemolysis and kidney damage, which can seriously affect animal growth performance [36]. Almost all of the unabsorbed copper is excreted in feces or urine, and may subsequently lead to environmental contamination [37–38]. In addition, there are no unified management measures for the treatment of animal feces, which is generally used as fertilizer and is in direct with the soil. Although, the mobility of metal in the soil is relatively low, it will gradually accumulate and cause pollution to the natural environment. The accumulation of copper has a significant impact on agricultural ecosystems and is thought to lead to a decline in soil fertility and water quality [39]. Some studies have suggested that excessive copper in the soil has a negative effect on soil microbial community function [40]. Furthermore, studies have also shown antagonism between trace elements (Cu and Zn, Fe, Mo, and S) in chicks and other animals [41,42]. The antagonism between trace elements is mainly due to the formation of insoluble complexes in the gastrointestinal tract by unexcreted metallic substances [42].

Through meta-analysis, this study attempts to synthesize existing research and provide a theoretical basis for the quantitative analysis of new copper demand. Meta-analysis is greatly influenced by the literature sources; however, the main sources of literature in this study are mainly from Chinese and English databases. Literature in other languages, papers in conference proceedings, and some unpublished results may have an impact on the meta-analysis. In addition, the number of studies included was limited. Although there was no publication bias in these studies, only one study was included in some subgroups, which does not lead to robust results. Thus, the results of this paper may have some limitations.

## 5. Conclusions

The demand for copper in modern broiler feeding far exceeds the NRC standard. Through mate-analysis, we found that the adjusted ADG, ADFI, and FCR showed a clear quadratic relationships with the amount of Cu added. The maximum ADG (31.84 g/d) was reached when 158 mg/kg of copper was added, and the minimum FCR (1.53) was reached when 217 mg/kg of copper was added. We suggest that the addition of copper in the broiler feeding process should be 217 mg/kg which may have a positive effect on broiler performance and improve economic benefits.

## Supporting information

S1 Checklist PRISMA 2009 checklist. (DOC)

S1 Table The characteristics of meta-analysis database. (DOCX)

S1 Fig PRISMA flow diagram. (DOC)

## References

[1] Luo XG, Ji F, Lin YX, Steward FA, Lu L, Liu B, et al Effects of dietary supplementation with copper sulfate or tribasic copper chloride on broiler performance, relative copper bioavailability, and oxidation stability of vitamin E in feed. Poult Sci. 2005; 84: 888–893. 10.1093/ps/84.6.888 15971525

[2] NRC Nutrient requirement for poultry, 9th ed; National Academies Press: Washington, USA, 1994; pp.62

[3] Leeson S. Copper metabolism and dietary needs. World Poult Sci J. 2009; 65: 353–366.

[4] Pesti GM, Bakalli RI. Studies on the feeding of cupric sulfate pentahydrate and cupric citrate to broiler chickens. Poult Sci. 1996; 75: 1086–1091. 10.3382/ps.0751086 8878264

[5] Ewing HP, Pesti GM, Bakalli RI, Menten JFM. Studies on the feeding of cupric sulfate pentahydrate, cupric citrate, and copper oxychloride to broiler chickens. Poult Sci. 1998; 77: 445–448. 10.1093/ps/77.3.445 9521458

[6] Arias VJ, Koutsos EA. Effects of copper source and level on intestinal physiology and growth of broiler chickens. Poult Sci. 2006; 85: 999–1007. 10.1093/ps/85.6.999 16776467

[7] Karimi A, Sadeghi G, Vaziry A. The effect of copper in excess of the requirement during the starter period on subsequent performance of broiler chicks. J Appl Poult Res. 2011; 20: 203–209.

[8] Konjufca V, Pesti G, Bakalli R. Modulation of cholesterol levels in broiler meat by dietary garlic and copper. Poult Sci. 1997; 76: 1264–1271. 10.1093/ps/76.9.1264 9276889

[9] Arias VJ, Koutsos EA. Effects of copper source and level on intestinal physiology and growth of broiler chickens. Poult Sci. 2006; 85: 999–1007. 10.1093/ps/85.6.999 16776467

[10] Persia ME, Baker DH, Parsons CM. Tolerance for excess basic zinc chloride and basic copper chloride in chicks. Brit Poult Sci. 2004; 45: 672–676. 15623222 10.1080/00071660400006172

[11] Sauvant D, Schmidely P, Daudin JJ, St-Pierre NR. Meta-analyses of experimental data in animal nutrition. Animal. 2008; 2: 1203–1214. 10.1017/S1751731108002280 22443733

[12] Gurevitch J, Hedges LV. Meta-analysis: combining results of independent experiments. New York: Chapman and Hall; 1993.

[13] St-Pierre NR. Integrating quantitative findings from multiple studies using mixed model methodology. J Dairy Sci. 2001; 84: 741–755. 10.3168/jds.S0022-0302(01)74530-4 11352149

[14] Pekel AY, Patterson PH, Hulet RM, Acar N, Cravener TL, Dowler DB, et al Dietary camelina meal versus flaxseed with and without supplemental copper for broiler chickens: live performance and processing yield. Poult Sci. 2009; 88: 2392–2398. 10.3382/ps.2009-00051 19834091

[15] Bartov I. Effects of propionic acid and of copper sulfate on the nutritional value of diets containing moldy corn for broiler chicks. Poult Sci. 1983; 62: 2195–2200. 10.3382/ps.0622195 6657562

[16] Ledoux DR, Miles RD, Ammerman CB, Harms RH. Interaction of dietary nutrient concentration and supplemental copper on chick performance and tissue copper concentrations. Poult Sci. 1987; 66: 1379–1384. 10.3382/ps.0661379 3684858

[17] Pesti GM, Bakalli RI. Studies on the feeding of cupric sulfate pentahydrate and cupric citrate to broiler chickens. Poult Sci.1996; 75: 1086 10.3382/ps.0751086 8878264

[18] Karimi A, Sadeghi G, Vaziry A. The effect of copper in excess of the requirement during the starter period on subsequent performance of broiler chicks. J Appl Poult Res. 2011; 20: 203–209.

[19] Liu S, Lu L, Li S, Xie J, Zhang L, Wang R, et al Copper in Organic Proteinate or Inorganic Sulfate Form is Equally Bioavailable for Broiler Chicks Fed a Conventional Corn-Soybean Meal Diet. Biol Trace Elem Res. 2012; 147: 142–148. 10.1007/s12011-012-9329-5 22281815

[20] Samanta B, Biswas A, Ghosh PR. Effects of dietary copper supplementation on production performance and plasma biochemical parameters in broiler chickens. Br Poult Sci. 2011; 52: 573–577. 10.1080/00071668.2011.608649 22029784

[21] Konjufca VH, Pesti GM, Bakalli RI. Modulation of cholesterol levels in broiler meat by dietary garlic and copper. Poult Sci. 1997; 76: 1264–1271. 10.1093/ps/76.9.1264 9276889

[22] Luo XG, Ji F, Lin YX, Steward FA, Lu L, Liu B, et al Effects of dietary supplementation with copper sulfate or tribasic copper chloride on broiler performance, relative copper bioavailability, and oxidation stability of vitamin E in feed. Poult Sci. 2005, 84(6): 888–893. 10.1093/ps/84.6.888 15971525

[23] Zhang ZJ, Lv L, Luo XG, Liu B. Effect of tribasic copper chloride on growth performance of broilers fed in floor pens, and stability of vitamin E and phytase in feeds. Acta Nutr Sin. 2008; 30: 470–474.

[24] Sun XQ, Wang YH, Tan J, Xing XM, Zhao YJ. Effects of copper, iron, zinc and manganese level on growth performance and immune organs development of broiler with 0–3 weeks age. China Feed. 2007; 13: 21–24.

[25] Zhang XQ. Effects of Dietary Copper Source and Copper Level on Performance, Shank Pigmentation and Tissues’ Nutrients Deposition in Broiler. M. Sc. Thesis, The Sichuan Agriculture University. 2009.

[26] Pesti GM, Bakalli RI. Studies on the effect of feeding cupric sulfate pentahydrate to laying hens on egg cholesterol content. Poult Sci. 1998, 77(10): 1540–1545. 10.1093/ps/77.10.1540 9776063

[27] Pang Y, Applegate TJ. Effects of dietary copper supplementation and copper source on digesta pH, calcium, zinc, and copper complex size in the gastrointestinal tract of the broiler chicken. Poult Sci. 2007; 86: 531–537. 10.1093/ps/86.3.531 17297166

[28] Chowdhury S, Paik I, Namkung H, Lim H. Responses of broiler chickens to organic copper fed in the form of copper-methionine chelate. Anim Feed Sci Technol. 2004; 115: 281–293.

[29] Ruiz JA, Pérez-Vendreli AM, Esteve-Garcia E. Effect of dietary iron and copper on performance and oxidative stability in broiler leg meat. Br Poult Sci. 2000; 41: 163–167. 10.1080/713654910 10890211

[30] Miles RD, O’Keefe SF, Henry PR, Ammerman CB, Luo XG. The effect of dietary supplementation with copper sulfate or tribasic copper chloride on broiler performance, relative copper bioavailability, and dietary provident activity. Poult Sci. 1998; 77: 416–425. 10.1093/ps/77.3.416 9521454

[31] Aggrey SE, Karnuah AB, Sebastian B, Anthony NB. Genetic properties of feed efficiency parameters in meat-type chickens. Genet Sel Evol. 2010, 42(1), 25. 20584334 10.1186/1297-9686-42-25 PMC2901204

[32] Fox MC, Brown DR, Lee LS. Effect of Dietary Buffer Additions on Gain, Efficiency, Duodenal pH, and Copper Concentration in Liver of *Eimeria acervulina-lnfected* Chicks. Poult Sci. 1987, 66 3: 500–504. 10.3382/ps.0660500 3601861

[33] Burnell TW, Cromwell GL, Stahly TS. Effects of dried whey and copper sulfate on the growth responses to organic acid in diets for weanling pigs. J Anim Sci.1988; 66: 1100–1108. 10.2527/jas1988.6651100x 3397336

[34] Yang W, Zhao C, Zhang C, Yang L. High Dietary Copper Increases Catecholamine Concentrations in the Hypothalami and Midbrains of Growing Pigs. Biol Trace Elem Res. 2016; 170: 115–118. 10.1007/s12011-015-0460-y 26263873

[35] Persia ME, Baker DH, Parsons CM. Tolerance for excess basic zinc chloride and basic copper chloride in chicks. Br poult Sci. 2004, 45(5): 672–676. 10.1080/00071660400006172 15623222

[36] Gaetke LM, Chow CK. Copper toxicity, oxidative stress, and antioxidant nutrients. Toxicology, 2003, 189(1–2): 147–163. 10.1016/s0300-483x(03)00159-8 12821289

[37] Bolan NS, Khan MA, Donaldson J, et al Distribution and bioavailability of copper in farm effluent. Sci Total Environ. 2003, 309(1–3): 225–236. 10.1016/S0048-9697(03)00052-4 12798106

[38] Nicholson FA, Smith SR, Alloway BJ, et al An inventory of heavy metals inputs to agricultural soils in England and Wales. Sci Total Environ. 2003, 311(1–3): 205–219. 10.1016/S0048-9697(03)00139-6 12826393

[39] Han FX, Kingery WL, Selim HM, et al Accumulation of heavy metals in a long-term poultry waste-amended soil. Soil Sci. 2000, 165(3): 260–268.

[40] Kong WD, Zhu YG, Fu BJ, et al The veterinary antibiotic oxytetracycline and Cu influence functional diversity of the soil microbial community. Environ Pollut. 2006, 143(1): 129–137. 10.1016/j.envpol.2005.11.003 16413090

[41] Southern LL, Baker DH. Zinc toxicity, zinc deficiency and zinc-copper interrelationship in *Eimeria acervulina*-infected chicks. J Nutr. 1983, 113(3): 688–696. 10.1093/jn/113.3.688 6827384

[42] Spears JW. Trace mineral bioavailability in ruminants. J. Nutr. 2003, 133(5): 1506S–1509S. 12730454 10.1093/jn/133.5.1506S

