# Supplementary material for: Meta-analysis of the correlation between dietary copper supply and broiler performance
Source: PLoS One. 2020 May 18;15(5):e0232876. doi: 10.1371/journal.pone.0232876 (PMC7233574; doi:10.1371/journal.pone.0232876)
Supplement: S1 Table — (DOCX) [file pone.0232876.s002.docx]

**S1 Table The characteristics of Meta-analysis database**

| **Author** | **Country** | **Published Year** | **Strains** | **Feedstuff** | **Copper Source** | **Dosage (mg/kg)** | **No. of samples** | **Experimental period (d)** |
| --- | --- | --- | --- | --- | --- | --- | --- | --- |
| Pekel [14] | US | 2009 | Cobb × Avian-48 | corn+soybean | CuSO4·5H20 | 150 | 294 | 21 |
| Bartov [15] | Israel | 1983 | Hubbard male | corn+soybean | CuSO4·5H20 | 150 | 405 | 21 |
| Ledoux [16] | US | 1986 | Cobb feather sexed female | corn+soybean | CuSO4·5H20 | 400, 800 | 576 | 21 |
| Pesti [17] | US | 1996 | Peterson × Arbor Acres | corn+soybean | CuSO4·5H20 or Cu proteinate | 125, 250, 375 | 360 | 21 |
| Karimi [18] | Iran | 2011 | Ross 308 | corn+soybean | CuSO4·5H20 | 125, 250 | 156 | 21 |
| Liu [19] | China | 2012 | Arbor Acres male | corn+soybean | CuSO4·5H20 | 125, 250 | 320 | 21 |
| Samanta [20] | India | 2011 | Vencobb-100 | corn+soybean | CuSO4·5H20 or TBCC | 75, 150, 250 | 240 | 21 |
| Konjufca [21] | US | 1997 | Ross × Ross 208 male | corn+soybean | CuSO4·5H20 or TBCC | 63, 180 | 208 | 21 |
| Luo [22] | China | 2005 | Arbor Acres male | corn+soybean | CuSO4·5H20 | 150, 300, 450 | 420 | 21 |
| Zhang [23] | China | 2006 | Arbor Acres male | corn+soybean | CuSO4·5H20 or TBCC | 100, 150, 200 | 840 | 21 |
| Sun [24] | China | 2007 | Avian | corn+soybean | CuSO4·5H20 | 3.25, 8, 12.75, 16 | 960 | 21 |
| Zhang [25] | China | 2008 | Cobb-48 | corn+soybean | CuSO4·5H20 or TBCC | 50, 150, 250, 350 | 270 | 21 |
