# Supplementary material for: Meta-analysis of the correlation between dietary copper supply and broiler performance
Source: PLoS One. 2020 May 18;15(5):e0232876. doi: 10.1371/journal.pone.0232876 (PMC7233574; doi:10.1371/journal.pone.0232876)
Supplement: S1 Fig — (DOC) [file pone.0232876.s003.doc]

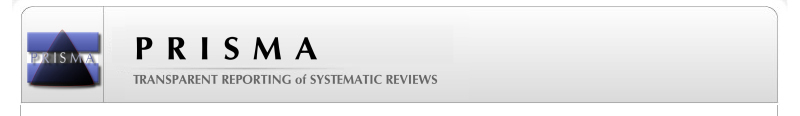
**PRISMA 2009 Flow Diagram**

**Screening**

**Included**

**Eligibility**

**Identification**

Records identified through database searching
(n =4271)

Additional records identified through other sources
(n =0)

Records after duplicates removed
(n = 4125)

Records screened
(n = 2167)

Records excluded
(n =1958)

Full-text articles assessed for eligibility
(n =113)

Full-text articles excluded, with reasons
(n =100)

Studies included in qualitative synthesis
(n =12)

Studies included in quantitative synthesis (meta-analysis)
(n =12)
